# Design, Implementation, and Evaluation of a Variable Stiffness Transradial Hand Prosthesis

**DOI:** 10.3389/fnbot.2022.789210

**Published:** 2022-03-10

**Authors:** Elif Hocaoglu, Volkan Patoglu

**Affiliations:** ^1^Faculty of Engineering and Natural Sciences, Sabancı University, Istanbul, Turkey; ^2^School of Engineering and Natural Sciences, Istanbul Medipol University, Istanbul, Turkey

**Keywords:** transradial hand prosthesis, underactuated robotic hand design, variable stiffness actuation, impedance modulation, tele-impedance control

## Abstract

We present the design, implementation, and experimental evaluation of a low-cost, customizable, easy-to-use transradial hand prosthesis capable of adapting its compliance. Variable stiffness actuation (VSA) of the prosthesis is based on antagonistically arranged tendons coupled to nonlinear springs driven through a Bowden cable based power transmission. Bowden cable based antagonistic VSA can, not only regulate the stiffness and the position of the prosthetic hand but also enables a light-weight and low-cost design, by the opportunistic placement of motors, batteries, and controllers on any convenient location on the human body, while nonlinear springs are conveniently integrated inside the forearm. The transradial hand prosthesis also features tendon driven underactuated compliant fingers that allow natural adaption of the hand shape to wrap around a wide variety of object geometries, while the modulation of the stiffness of their drive tendons enables the prosthesis to perform various tasks with high dexterity. The compliant fingers of the prosthesis add inherent robustness and flexibility, even under impacts. The control of the variable stiffness transradial hand prosthesis is achieved by an sEMG based natural human-machine interface.

## 1. Introduction

Versatile grasping and manipulation in unstructured environments are challenging tasks, actively investigated in robotics. Multi-fingered robot hands have been developed both in academia (Dalley et al., 2009; Takaki and Omata, 2011; Chen et al., 2015) and for commercial use (Liberating Technologies Inc., 2022; Touch Bionics Inc., 2022) to achieve various tasks. Anthropomorphism (ability to emulate human-like hand shape, size, and consistency) and dexterity (successful manipulation capability even under unstructured conditions) are commonly identified as the key features to reach a satisfactory level of performance.

Anthropomorphism is an important criterion in the design of robotic end-effectors, especially for the purpose of hand prostheses (Bicchi, 2000), since the tools around the environment, such as consoles, handles, keys, are designed for the human hands. In addition, anthropomorphic designs are aesthetically and physiologically more fulfilling for amputees, as they provide natural appearances. However, anthropomorphism alone is not sufficient; other important criteria, such as simple but robust design, ease of use, and adequate level of dexterity are also crucial factors in the design of prosthetic hands.

Dexterity is a quite evident goal for the robotic and prosthetic hands in order for them to be endowed with human-like capabilities, such as grasping objects and performing fine finger movements for precise manipulations. In order for a prosthetic hand to qualify as a dexterous design, it has to be capable of performing most of the human hand taxonomy required during the activities of daily living (ADL) (Feix et al., 2016). In the literature (Bicchi et al., 2011; Catalano et al., 2012), it has been emphasized that the majority of human grasps are power grasps, which is preferred for more than 50% of the time when the hand is used. Pinch grasp is ranked as second with a 20% preference rate (Feix et al., 2016). Hence, since power grasps and pinch grasps are the most common hand functions, providing hand prostheses with these dominant grasp types may be prioritized to execute most ADL.

Successful manipulation necessitates another significant and commonly neglected characteristic of the human hand, namely impedance modulation. Incorporating impedance modulation property in the design of a hand prosthesis makes it adaptable to interacted objects/tasks. Successful execution of many ADLs, where human physically interacts with the environment, arises from the proper modulation of the impedance level of hand based on the varying requirements of the task. For instance, some activities, such as writing and painting, necessitate highly accurate position control for which the stiffness of the fingers is increased considerably, while manipulation of soft/fragile objects, such as holding an egg or picking up an apricot, requires low stiffness of the fingers.

The impedance modulation property of human hands has inspired design and control strategies for robotic and prosthetic hands, whose goal is to improve the quality of interaction with dynamic environments, especially under unpredictable conditions. Specifically, hand prostheses become safer and more functional if the appropriate impedance level based on the physical conditions of the interacted environment can be ensured (Abul-Haj and Hogan, 1987, 1990; Mengilli et al., 2020; Tosun and Patoglu, 2020). Several studies (Blank et al., 2011, 2012, 2013; Hocaoglu, 2014; Kara and Patoglu, 2020) provide strong evidence that hand prostheses with stiffness modulation can improve the performance of an amputee when the impedance of the prosthesis is matched to the requirements of the task.

Impedance modulation approaches in robotics can be grouped into two major categories. In the first category, the compliance of the device is modulated through software, using strategies such as impedance/admittance control. In this approach, the impedance modulation is limited by the controllable bandwidth of the actuators, and for this reason, a prosthesis whose impedance is modulated with such a control strategy behaves like a rigid body for high frequency excitations, such as impacts that exceed its control bandwidth (Abul-Haj and Hogan, 1990; Mengilli et al., 2020; Tosun and Patoglu, 2020). Moreover, this approach requires continuous use of actuators and suffers from low energy efficiency.

In the second category, impedance modulation is embedded into the mechanical design of a robotic system. In this approach, the impedance of the robotic manipulator is adjusted through special mechanisms consisting of passive elastic elements, such as springs. In hardware based impedance modulation, e.g., variable stiffness actuation (VSA), the impedance change is physical and is valid for the whole frequency spectrum, including frequencies well above the controllable bandwidth of the actuators. Furthermore, this approach consumes energy only when the impedance is being modulated; hence, is energy efficient.

Achieving human level dexterity with fully actuated, high degrees of freedom prosthetic hands requires the use of complex control algorithms (Touvet et al., 2012). In the literature, there exists myoelectrically controlled hand prostheses that are capable of performing a large variety of manipulation tasks (Chu et al., 2007; Castellini et al., 2008; Li and Kuiken, 2009); however, the control of each finger joint in these systems is realized by means of sophisticated algorithms, whose complexity exposes amputees to long training periods. The burden of long training periods and complexity involved in controlling prosthetic devices are known to contribute to a high abandonment rate for these devices, reaching up to 40% (McFarland et al., 2010). A large percentage of amputees reject active prostheses since they are dissatisfied with the current level of the functionality provided by these devices, given their complexity. These amputees prefer easy to use passive prostheses, even at a cost of reduced functionality (Biddiss and Chau, 2007). To address the challenges of low adaptation and high abandonment rate of active prostheses, several research groups have focused on the simplification of mechanical design and ensuring ease of control, without losing the main functionality of prosthetic hands.

In the literature, several robotic hands employed for tasks requiring human machine interaction have been designed using VSA (Grebenstein et al., 2010; Wolf et al., 2011; Shadow Robot Company Ltd., 2022), while, to the best of authors' knowledge, no such application has been reported in the field of anthropomorphic hand prostheses. In particular, each active degree of freedom of the DLR Hand Arm System is controlled by two motors attached to antagonistically arranged nonlinear spring elements (Grebenstein et al., 2010; Wolf et al., 2011). Similarly, the impedance modulation of the anthropomorphic Shadow Hand is achieved by antagonistically arranged pneumatic artificial muscles (Shadow Robot Company Ltd., 2022). Both DLR Hand Arm System and Shadow Hand feature sophisticated mechanical designs with a large number of active degrees of freedom; hence, their size, weight, and cost make them infeasible for use as a hand prosthesis. Furthermore, grasp planning and impedance modulation of these devices necessitate complex algorithms, which renders their use quite challenging.

The employment of underactuated mechanisms for hand designs is a promising approach, as underactuated hands have been shown to provide a remarkable adaptation to various object geometries without the need for sensors or complex control algorithms. Underactuation is commonly implemented by either linkage or tendon based finger designs that are capable of performing typical human-like finger closing sequences. Linkage based underactuated fingers (Birglen and Gosselin, 2003; Birglen et al., 2008; Kamikawa and Maeno, 2008; Prattichizzo et al., 2012; Ertas et al., 2014) are capable of shape adaptation and can endure larger forces compared to tendon based ones; however, their relatively bulky design makes them not well-suited for integration into anthropomorphic prosthetic hands. Most successfully implementations of anthropomorphic underactuated hand designs have been realized by means of tendon driven mechanisms (Massa et al., 2002; Gosselin et al., 2008; ya Nagase et al., 2011; Xu et al., 2015), since slim and lightweight fingers can be actuated with this method. For instance, in Dollar and Howe (2006), Dollar et al. (2010), and Ma et al. (2013), a compliant, underactuated, sensor integrated robotic hand with tendon driven elastic joints is introduced, and fabricated *via* support decomposition manufacturing. Moreover, in Godfrey et al. (2018), an underactuated hand with a single motor is designed to adapt to objects with different geometric shapes by means of its tendon driven elastic joints. With its underactuated finger mechanism and elastic joints, this low cost hand is capable of self-adaptation to different shaped objects under simple control methods. Similarly, a tendon driven underactuated robot hand that explores synergies of human hand motions is implemented in Catalano et al. (2014). However, neither of these underactuated hands feature impedance modulation capabilities.

In this study, we present the design, fabrication, and evaluation of a variable stiffness transradial hand prosthesis to be controlled through a natural human-machine interface. VSA of the prosthesis is based on antagonistically arranged tendons coupled to nonlinear springs driven through a Bowden cable based power transmission. Bowden cable based antagonistic VSA regulates both the impedance and the position of the hand. It also enables a light-weight hand design, by opportunistically placing the motors, batteries, and controllers to any convenient location on the human body, while nonlinear springs are conveniently integrated inside the forearm. The proposed prosthesis features tendon driven underactuated compliant fingers that enable natural adaption of the hand shape to wrap around a wide variety of object geometries and modulation of the hand's stiffness to perform various tasks with high dexterity. The compliant fingers are built from polyurethane with a low-cost manufacturing process and add inherent robustness and flexibility, even under unexpected conditions such as impacts.

The control of the variable stiffness transradial hand prosthesis is achieved by a natural human-machine interface that utilizes sEMG signals measured from the surface of the upper arm, chest, and shoulder. This natural control interface, called *tele-impedance controller*, is first presented in Hocaoglu and Patoglu (2012), while the detailed implementation of this controller and its performance evaluation are presented in Hocaoglu (2014) and Hocaoglu and Patoglu (2019).

The rest of the manuscript is organized as follows: Section 2.1 introduces the design objectives for the variable stiffness transradial hand prosthesis, while Section 2.2 reviews its sEMG-based tele-impedance control. Section 2.3 details the mechatronic design of the VSA prosthesis. Section 3 presents experimental evaluations that provide evidence of the working principle. Section 3.3 evaluates the grasping performance of the hand prosthesis with a wide variety of objects and provides a discussion of the results. Finally, Section 4 concludes the article and discusses future studies.

## 2. Materials and Methods

This section presents the design objectives for the VSA hand prosthesis, overviews its sEMG-based control approach, and implementation of the proposed objectives.

### 2.1. Design Objectives

Following the terminology in Merlet (2006), one can categorize the performance requirements for hand prostheses into four groups: imperative, optimal, primary, and secondary requirements.

Anthropomorphism is an imperative design requirement for hand prostheses. Aesthetically pleasing natural appearance is not only necessary for the adaptation of prosthetic devices by amputees, but also anthropomorphic designs are better suited to interact with common human-oriented tools and environments. In this study, we design an anthropomorphic hand prosthesis, for which the dimensions are customizable.

Dexterity is an optimal performance requirement that needs to be maximized while designing a hand prosthesis. In particular, a hand prosthesis should be capable of grasping objects with different shapes (prismatic, spherical, cylindrical) and with various properties (soft or fragile structures, smooth or ragged surfaces) without damaging them. In this study, we ensure dexterity by designing an underactuated hand that can mimic the opening/closing sequence of human fingers to adapt to a wide variety of geometries and by enabling the stiffness of the prosthesis to be actively modulated based on the task.

The primary requirement for a hand prosthesis is ease-of-use. The control of the device should be intuitive, allowing amputees to use the device without being exposed to long training periods. Moreover, the hand prosthesis should be energy efficient and its batteries should be easily swappable for user friendliness. In this study, the use of an underactuated design simplifies the control of the device, as only the position and impedance of the drive tendon need to be controlled. The tele-impedance controller reviewed in Section II-B provides a natural sEMG interface for the control of the device, where the impedance modulation takes place automatically, allowing the amputee only to focus on the position control of the hand. Energy efficiency is ensured by hardware based impedance modulation, where no energy is wasted to maintain a desired impedance level. Finally, Bowden cable based actuation enables batteries to be opportunistically placed anywhere on the body, making them easily re-sizeable or swappable.

The secondary requirements for the device are high robustness and low cost. In this study, the compliant fingers and the VSA provide built-in physical compliance that provides inherent robustness to impacts. Besides, all parts of the prosthesis are simple to manufacture and customizable. Furthermore, since Bowden cable based actuation allows for motors, drivers, and batteries of the system to be remotely located, the cost of these parts can be kept low, as strict size and weight constraints do not apply to these parts.

### 2.2. Overview of sEMG-Based Control Architecture

**Figure 1:**
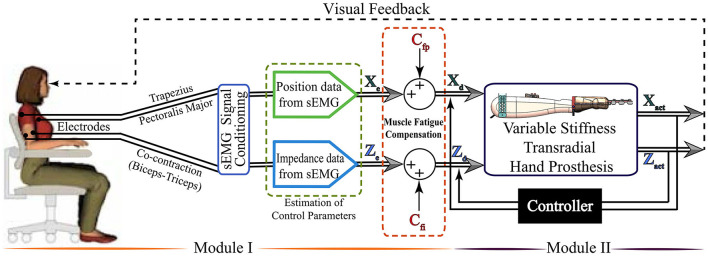
Tele-impedance control architecture of the variable stiffness transradial hand prosthesis.

The tele-impedance control architecture consists of two modules, as depicted in Figure 1. The first module handles the measurement of sEMG signals, their conditioning, and the estimation of reference values for the hand position and stiffness. The second module implements a closed-loop controller that ensures that the position and the stiffness of the VSA prosthetic hand match the reference values. Throughout the control, visual feedback and physical coupling provide information to the amputees to adapt their sEMG signals to match the requirements of the task.

Given that transradial upper extremity amputees lack the muscle groups responsible for hand and forearm motions, sEMG signals for the position control of the hand prosthesis are measured from chest and shoulder muscles, while sEMG signals used for stiffness regulation are measured from the muscle pairs on the upper arm. The estimation of the hand position and stiffness from sEMG signals involves modeling of hand motion/stiffness based on sEMG signals, empirical determination of the parameters of these models for use in real time control, and incorporation of fatigue compensation.

Conditioned sEMG signals are discretized into several levels to map them to the physical stiffness range of the VSA and the range of motion of the fingers. A calibration procedure is performed for sEMG signals before each use, to customize the maximum voluntary contraction (MVC) levels for the user, as commonly done for commercial prosthetic hands. Since the MVC levels of the sEMG signals vary in time and are specific to users, the sEMG signals have to be normalized before each use to present accurate reference data to the controller shown in the second module in Figure 1. Accordingly, the required time to calibrate the sEMG signal for every initiation of the hand prosthesis is around 3 min. The first 30 s is required to gather MVC data to normalize the signal. The rest of the duration is necessary to estimate the parameters for stiffness estimation. The device sequentially records the sEMG signals of the subject while performing the specific tasks and does the implementation automatically before activating the device for the subjects. There is around a 15 s resting period between sEMG data recordings to prevent the fatigue effect in the calibration data.

The sEMG based tele-impedance control interface, together with the VSA, enables an amputee to modulate the stiffness of the prosthetic hand to properly match the requirements of the task while performing ADL under visual feedback. The regulation of stiffness is managed through the stiffness measurements of the intact upper arm, and this control takes place automatically as the amputee interacts with the environment. The position of the hand prosthesis is controlled intentionally by the amputee through the position of the shoulder, estimated using SEMG signals. This natural human-robot interface is advantageous since the impedance regulation takes place naturally without requiring amputees' attention and diminishing their functional capability. Consequently, the proposed interface does not require long training periods or interfere with the control of intact body segments and is easy to use. Details of the controller and its experimental verification are presented in Hocaoglu and Patoglu (2019).

### 2.3. Design of Variable Stiffness Transradial Hand Prosthesis

To satisfy the design objectives, an anthropomorphic, VSA integrated, underactuated, compliant hand prosthesis is developed as follows.

#### 2.3.1. Bowden Cable Driven Antagonist VSA

An antagonistic VSA is utilized to control the position and the stiffness of a four-fingered, underactuated, compliant prosthetic hand. Tendon based antagonistic arrangement is preferred since it allows for the elastic elements and actuators to be conveniently placed away from the fingers of the prosthesis. Furthermore, VSA is driven by a Bowden cable based transmission that enables motors, drivers, and batteries to be located at any suitable place on the body of the amputee. This not only results in a light-weight design but also enables easy customization of these parts, e.g., force output or battery capacity, for any user.

##### 2.3.1.1. Implementation of Antagonistic VSA Using Expanding Contour Cams

**Figure 2:**
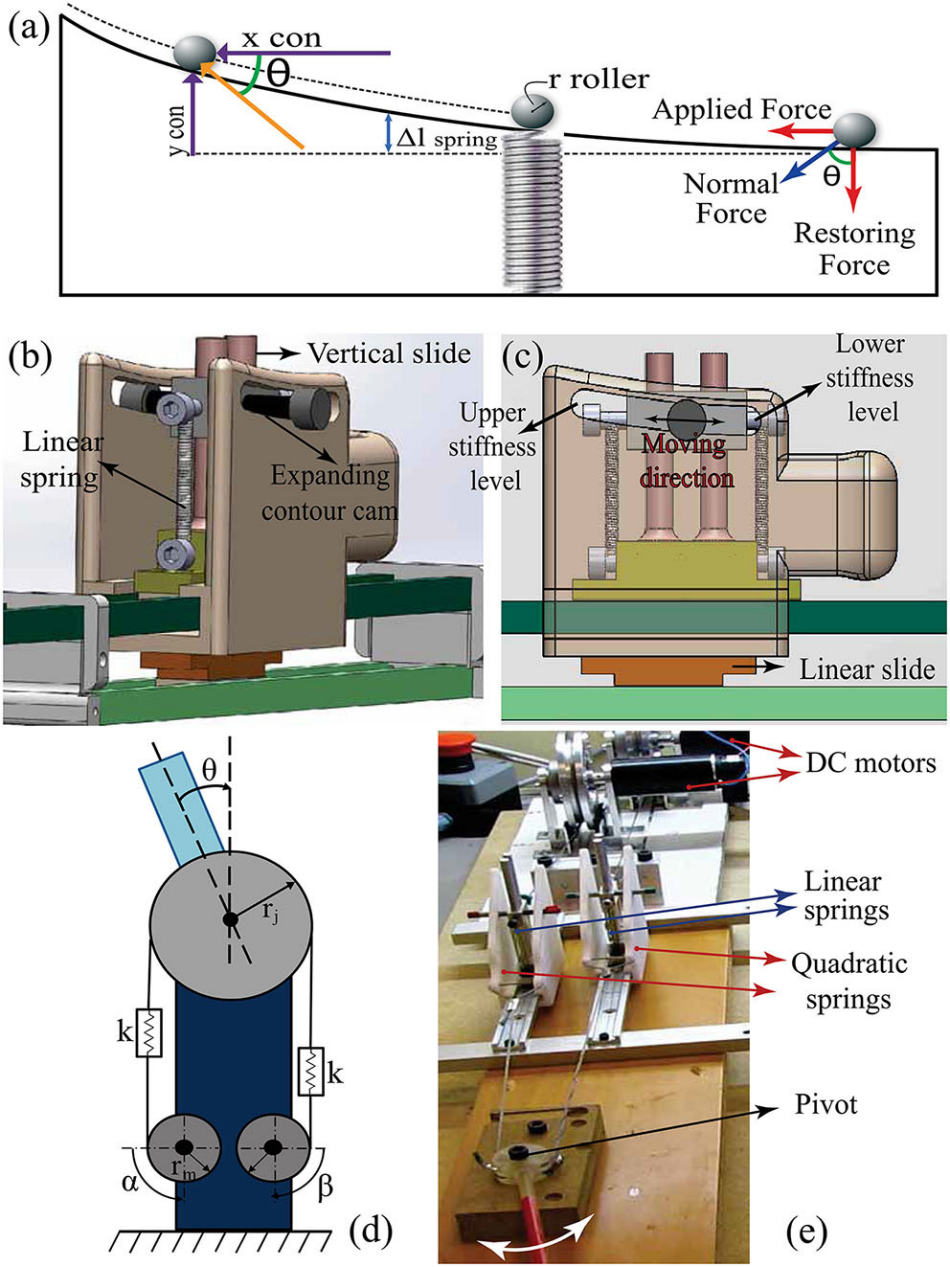
**(a)** The expanding contour cam, **(b)** solid model of the antagonistic variable stiffness actuation (VSA), **(c)** components of VSA, **(d)** schematic model of the antagonistically driven VSA, and **(e)** the experimental set-up used to verify the VSA.

It is well-established that an antagonistic VSA can mimic the independent stiffness and position control of a human limb joint under quasi-static conditions if the antagonistic spring elements of the VSA have nonlinear (typically quadratic) deflection-force characteristics (English and Russell, 1999). One way of attaining the desired nonlinear spring relationship is to utilize linear springs constrained to move on nonlinear expanding surfaces, called expanding contour cams (Migliore et al., 2007). In such an arrangement, as shown in Figures 2a–c, when the force is exerted on the system, linear springs extend according to the nonlinear cam surface; hence, a nonlinear relationship between the spring force and the deflection is ensured. The expanding contour cams implement the gradient of the force-deflection relationship and can be designed based on the linear spring constant and the maximum-minimum joint stiffness values.

Since the VSA aims at modulating the stiffness of prosthetic fingers, the design is implemented based on the maximum and the minimum joint stiffness values of human fingers, as given in Howe et al. (1985). These two design parameters along with the linear spring constant are sufficient to characterize the shape of the expanding contour as shown below. Different from the design in Migliore et al. (2007), our expanding contour cams are designed to be single sided, such that they are compact enough to be integrated into the forearm portion of the prosthesis. Furthermore, the springs on single sided cams are mounted on vertical slides, enabling easy connections, providing stable movements, and preventing the linear springs from bending.

To simplify the design process, the number of parameters required to determine the expanding cam profile is reduced as follows. The free lengths of the springs are selected such that the preload on the linear springs can be set to zero at the thinnest portion of the cam. Since the radius of the rollers is significantly small compared to the cam profile, their effect is neglected in the nonlinear contour equation. Consequently, the force-displacement relationship of elastic elements is chosen to satisfy the quadratic equation


(1) $$\begin{array}{l} F_{app}=ax_{con}^{2}+bx_{con}+c \end{array}$$

where *F*_*app*_ represents the applied force to the roller, *x*_*con*_ is the current position of the roller along the *x*-axis of the expanding contour, and *a*, *b*, and *c* are the coefficients of the quadratic equation. Along the *y*-axis, the following relationship holds for the linear springs


(2) $$\begin{array}{l} F_{restoring}=k\;y_{con} \end{array}$$

where *F*_*restoring*_ denotes the force applied by the linear spring on the cam along the *y*-axis, *y*_*con*_ is the current position of the roller along the *y*-axis of the expanding contour, and *k* is the spring constant of linear springs. Neglecting the frictional effects, the cam profile enforces a geometric relation between *F*_*app*_ and *F*_*restoring*_ that can be expressed as


(3) $$\begin{array}{c} y_{con}^{2}-\left(\frac{2a}{3k}\right)x_{con}^{3}-\left(\frac{b}{k}\right)x_{con}^{2}-\left(\frac{2c}{k}\right)x_{con}-m=0 \end{array}$$

Enforcing the following boundary conditions when the linear spring is at the initial point of the expanding contour


(4) $$\begin{array}{l} x_{con}=0,\;y_{con}=0 \end{array}$$

implies that *m* = 0. In Equation (3), the parameters required to design expanding contour are *a*, *b*, *c* and the spring constant *k* of the linear springs. It can be shown that *a*, *b*, and *c* are directly dependent on the maximum *S*_*max*_ and the minimum *S*_*min*_ stiffness values of the VSA as follows (English and Russell, 1999):


(5) $$\begin{array}{l} F_{app}=\underset{a}{\underset{︸}{\left(\frac{S_{max}-S_{min}}{4r_{j}^{2}\Delta x_{max}}\right)}}x_{con}^{2}+\underset{b}{\underset{︸}{\left(\frac{S_{min}}{2r_{j}^{2}}\right)}}x_{con}\\ \;-\underset{c}{\underset{︸}{\left(\frac{\Delta x_{max}(S_{max}^{2}-2S_{min}^{2})}{8r_{j}^{2}(S_{max}-S_{min})}\right)}} \end{array}$$

where *r*_*j*_ denotes the radius of the pulley used to implement the VSA and Δ*x*_*max*_ symbolizes the maximum deflection of the linear springs. When the linear springs are unstretched (*x*_*con*_ = 0), the joint stiffness level is regulated to its minimum level *S*_*min*_. In addition to this, when the linear springs reach their maximum stretch (*x*_*con*_ = *x*_*max*_), the joint stiffness is regulated at its maximum level *S*_*max*_.

##### 2.3.1.2. Position and Stiffness Control With Antagonist VSA

The position and the stiffness of the VSA are controlled through position control of Bowden cables driven by two geared DC motors. Figure 2d presents a schematic representation of the VSA. Let α and β denote the angular position of DC motors, while *S* and θ represent joint stiffness and angle, respectively. As a result of the physical compliant elements in its mechanical design, the VSA is advantageous as it alleviates the need for force/impedance control and allows robust motion control of actuators to be utilized to achieve high performance interaction control.

Under quasi-static conditions (English and Russell, 1999; Migliore et al., 2007), the equilibrium position θ and stiffness *S* of the VSA can be calculated as


(6) $$\begin{array}{lll} \theta & = & \frac{r_{m}}{2r_{j}}\left(\alpha -\beta \right)-\frac{\tau_{load}}{2r_{j}^{2}\left(ar_{m}\left(\alpha +\beta \right)+b\right)} \end{array}$$

(7) $$\begin{array}{lll} S & = & 2ar_{m}r_{j}^{2}\left(\alpha +\beta \right)+2br_{j}^{2} \end{array}$$

where *r*_*m*_ represents the radius of the pulleys attached to the geared DC motors, while the external torque applied to VSA is denoted by τ_*load*_.

When control references belonging to the joint position and stiffness are provided, the desired motor positions are computed from Equations (6), (7) and the motors are motion controlled to these values.

##### 2.3.1.3. Experimental Verification of VSA

**Figure 3:**
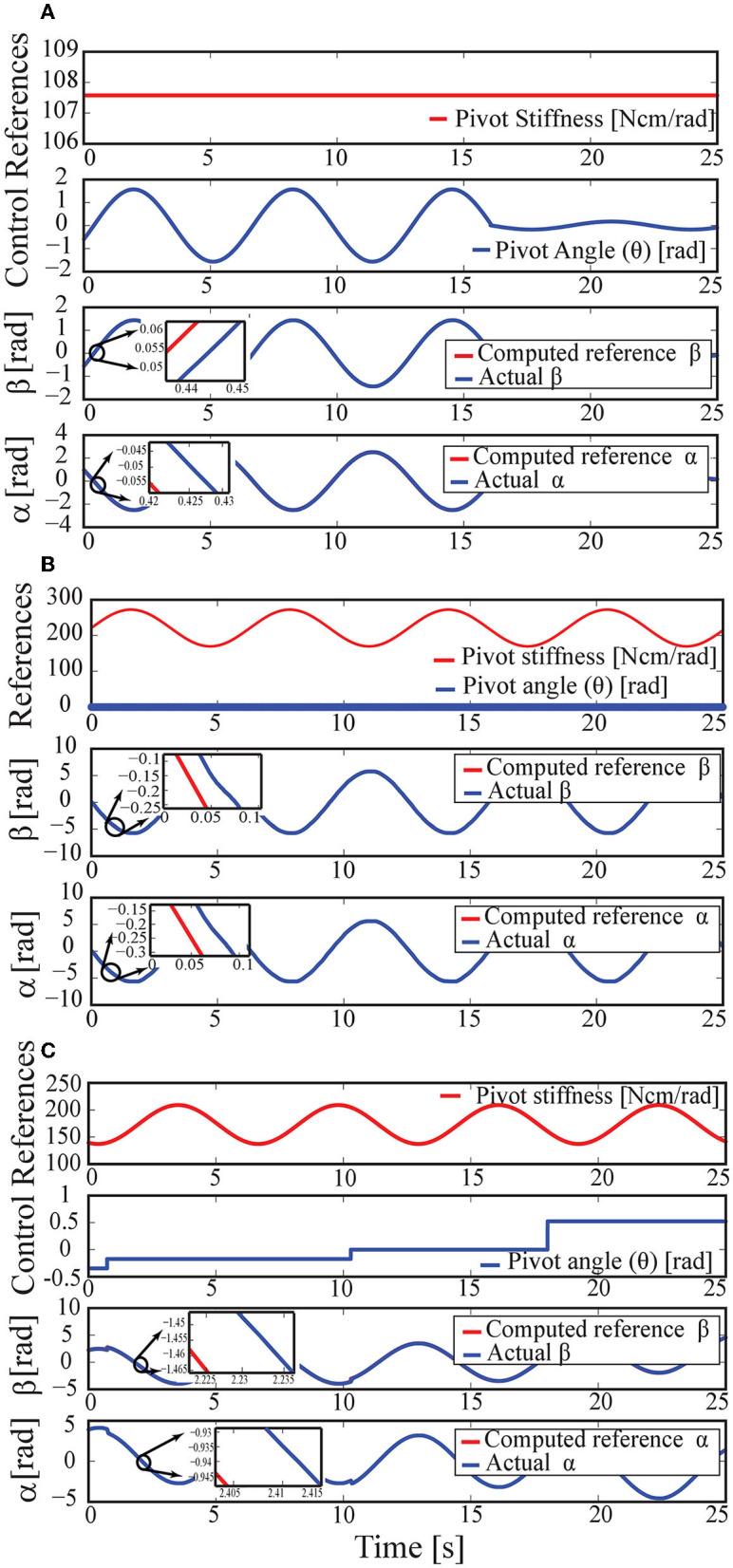
**(A)** Response to a sinusoidally changing position reference when the stiffness is kept constant. **(B)** Response to a sinusoidally changing stiffness reference when the position is kept constant. **(C)** Response to a sinusoidally changing stiffness while the position reference is gradually increased.

To experimentally verify the control performance of VSA, an antagonistic VSA is implemented using expanding contour cams shown in Figures 2b,c and is connected to a simple pivot. The aim of this experiment is to verify the independent and simultaneous position and stiffness control of the pivot *via* VSA. Several conditions are tested using the set-up shown in Figure 2e to evaluate the performance of VSA under realistic conditions. In particular, the following three conditions are evaluated sequentially: i) the position of the pivot is kept stationary while its stiffness is changed, ii) the stiffness of the pivot is kept constant while its position is modulated, and iii) both the position and the stiffness are varied simultaneously. Figures 3A–C present sample results from these experiments.

In Figure 3A, the pivot stiffness is kept at a constant level, while the position is simultaneously changed to track a sinusoidal reference of ±π/2 rad amplitude for the first 16 s and ±π/18 rad amplitude for the rest of the experiment. The results indicate that the position tracking RMS error of the motors is less than 0.03%.

Figure 3B presents results when the pivot stiffness is changed sinusoidally between the intermediate to the high level, while the angular position of VSA is kept constant at zero. The results indicate that the position tracking RMS error of the motors is less than 0.6%.

Figure 3C presents the response of the VSA to a sinusoidally changing stiffness reference, while the position reference is gradually increased with step changes. The results verify that the position tracking RMS error of the motors is less than 0.04%.

Robust motion controllers implemented with high gains and at high control rates keep the motion tracking error of the motors low, indicating that the estimated stiffness and position of the pivot can be controlled with good performance, even under the high friction forces induced by the Bowden cables. While the estimated stiffness and position of the pivot can be controlled with high precision, there exist other sources of errors, such as unmodelled dynamics of the VSA (the control model is valid only under quasi-static conditions) and the elasticity of the cable. The experimental results indicate that the position and stiffness tracking the performance of VSA are sufficiently high for use in a prosthetic hand, given that the position and the stiffness references will be provided in an amputee-in-the-loop fashion (under visual feedback) and discrete reference levels are required as discussed in Section 2.2. In particular, precise control of these values is not crucial, as evidence in the literature suggests that even a few discrete (e.g., low, moderate, and high) levels of stiffness can significantly improve the performance of typical manipulations (Blank et al., 2011, 2012, 2013; Hocaoglu, 2014; Kara and Patoglu, 2020).

#### 2.3.2. Underactuated Power Transmission

The proposed hand prosthesis is designed to feature underactuated power transmission, as a means of providing passive adaptation to various object geometries. Underactuation is preferred as it provides an ideal compromise between dexterous hands that provide versatile and stable grasps at high costs and computational loads, and simple grippers that excel at achieving specific tasks robustly with simple controllers at low costs but provide a relatively small set of grasps. Underactuation is the preferred alternative, since reducing the number of actuators required to control the system, not only saves weight, space, and cost but also provides energy efficiency and ease of control.

##### 2.3.2.1. Implementation of Underactuated Power Transmission

**Figure 4:**
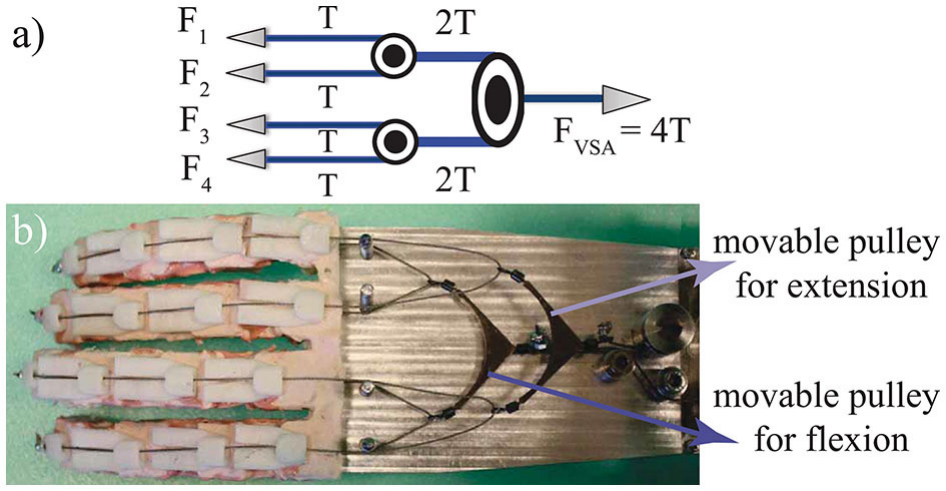
**(a)** Schematic representation of the pulley based transmission that equally distributes tendon tension to each finger when no external force is acting on the fingers. **(b)** Antagonistic actuation of the hand prosthesis.

The proposed hand prosthesis is designed to be actuated by a single VSA, such that the flexion/extension and the stiffness of fingers are controlled through antagonistic drive tendons. A pulley based power transmission, as in Figure 4a, distributes tendon tension to each phalanx based on the interaction forces.

When no external force is applied, the tendon tension is transferred equally to each of the four fingers, where each finger is composed of three compliant joints. The center of the pivot of VSA can be attached directly to the tendon that flexes the fingers to achieve unidirectional functionality for the hand, where the opening of the hand is performed by passive springs. We implement an alternative, where antagonistic springs of VSA are attached to the center of a moveable pulley mechanism, as shown in Figure 4b. In this arrangement, one of the movable pulleys transmits forces to flex the fingers, while its companion pulley transmits forces to extend them.

##### 2.3.2.2. Experimental Evaluation of the Underactuated Power Transmission

The natural grasping behavior of the underactuated prosthesis is tested over different shaped objects. Sample pinch and power grasps are presented in **Figure 10**.

As a result of underactuated power distribution, the compliant fingers naturally adapt to the shape of the objects to ensure that tendon tensions in each finger are equally distributed. That is, each finger seeks appropriate contact with the object (or a joint limit) to ensure proper force distribution of the tendons. As expected, the grasp type and the motion of each finger depending on the shape of the object and the relative configuration of the prosthesis.

#### 2.3.3. Design of Compliant Fingers

The proposed hand prosthesis is designed to feature compliant underactuated fingers. Compliant construction results in physical flexibility of the fingers, increasing their adaptability to the environment and robustness toward impacts. Underactuated kinematics with three compliant joints per finger increases the dexterity of the hand, by allowing it to wrap around a wide variety of objects. Underactuation also enables size, weight, and cost reduction for each finger, since the actuators are typically the largest, heaviest, and most expensive components of the device.

The underactuated compliant fingers are designed to mimic the closing sequence of human fingers, such that a coordinated motion of the phalanges is achieved. In particular, the stiffness of each compliant joint is adjusted such that they maintain the second and third phalanges of the finger in the fully extended configuration until the first phalanx comes in contact with an obstacle or reaches its mechanical limit. When the mechanism is free of contacts and within joint limits, it behaves like a single rigid body. When the motion of a phalanx is resisted, the force generated by the tendon overcomes the spring preload, and the adjacent phalanx initiates motion. The motion continues sequentially until movements of all phalanges are resisted, due to either contacting with an object or reaching the joint limits. Hence, each compliant finger is capable of producing many of the natural finger trajectories of a human hand, and the tendon force is properly distributed over all phalanges.

##### 2.3.3.1. Material Selection

The selection of appropriate material to cover the rigid phalanges and compliant joints is important to achieve robust fingers with a soft delicate touch. Selection of a high viscous silicon rubber results in high force requirements from the tendon and increases the energy consumption during bending, while the selection of a less viscous silicon rubber causes easy cracking of the material, decreasing the robustness of the finger. After testing many different materials, SILASTOSIL® 28-700 FG is evaluated to provide the best compromise among polyurethane materials to serve as the base material for the underactuated compliant fingers.

Implementation of proper stiffness for the compliant joints plays a crucial role in achieving the desired coordinated motion of the anthropomorphic fingers. Phalanges are fabricated with ABS plastic through rapid prototyping, while the compliant joints are fabricated using polyurethane material. Even though the polyurethane material is proper to serve as the base material to cover the compliant fingers, including the phalanges and compliant joints, it is necessary to further adjust the stiffness of each compliant joint to implement the desired coordinated motion and to anisotropically strengthen these joints against twisting and bending. Along these lines, carbon fiber strips are embedded inside each compliant joint. Lightweight carbon fiber strips not only act as leaf springs used to implement the desired level of compliance at each joint but also help support the joints by structurally reinforcing them against twisting and undesired bending forces.

##### 2.3.3.2. Fabrication of the Compliant Fingers

**Figure 5:**
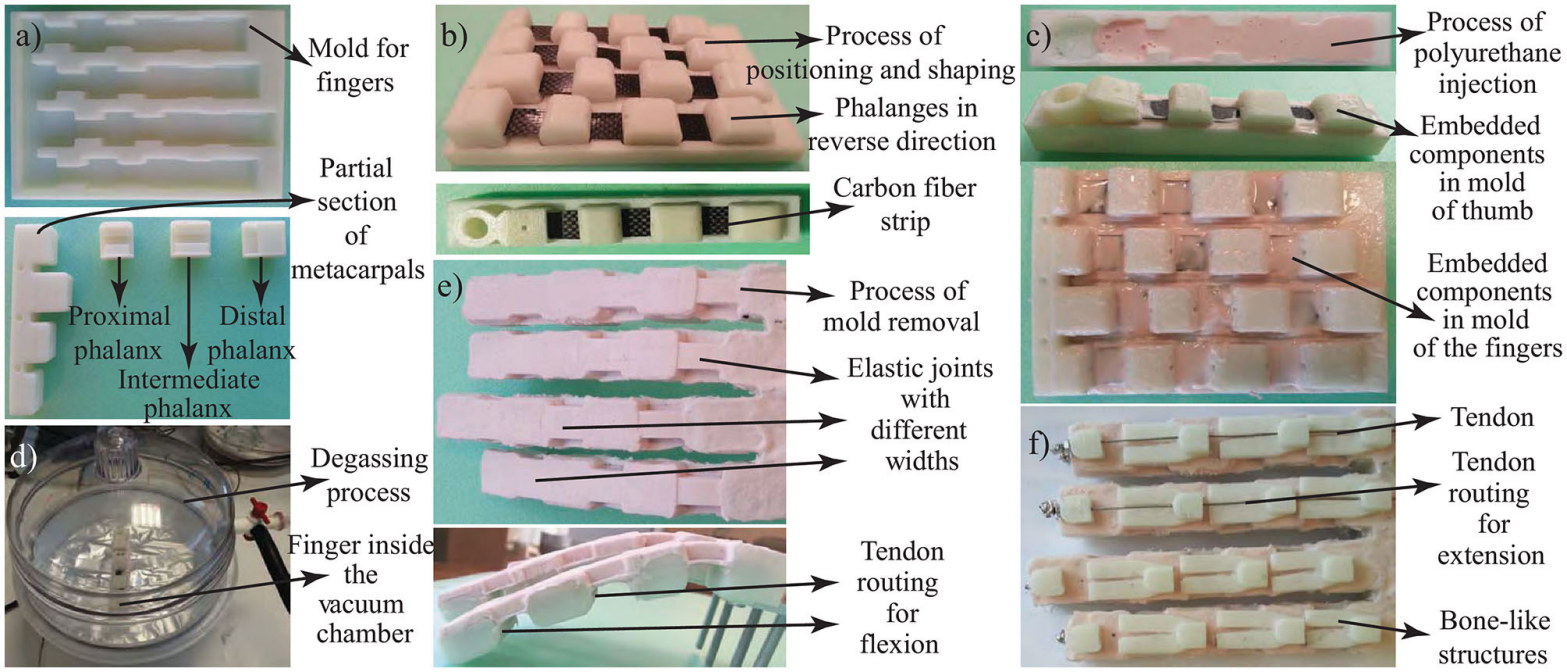
The six step process used to fabricate the compliant fingers. **(a)** Mold. **(b)** Positioning of the fingers. **(c)** Injection of the liquid polyurethane. **(d)** Degassing process. **(e)** Mold removal process. **(f)** Assembly of the other components.

Fabrication of compliant fingers consists of several stages as presented in Figure 5. In the first step, rigid parts, such as parts of phalanges and molds, are fabricated using additive manufacturing as shown in Figure 5a. Additive manufacturing enables the intricate design of phalanges and finger molds to be custom built for each amputee. Furthermore, accuracies of 100 μm are easily achievable at low manufacturing costs.

**Figure 6:**
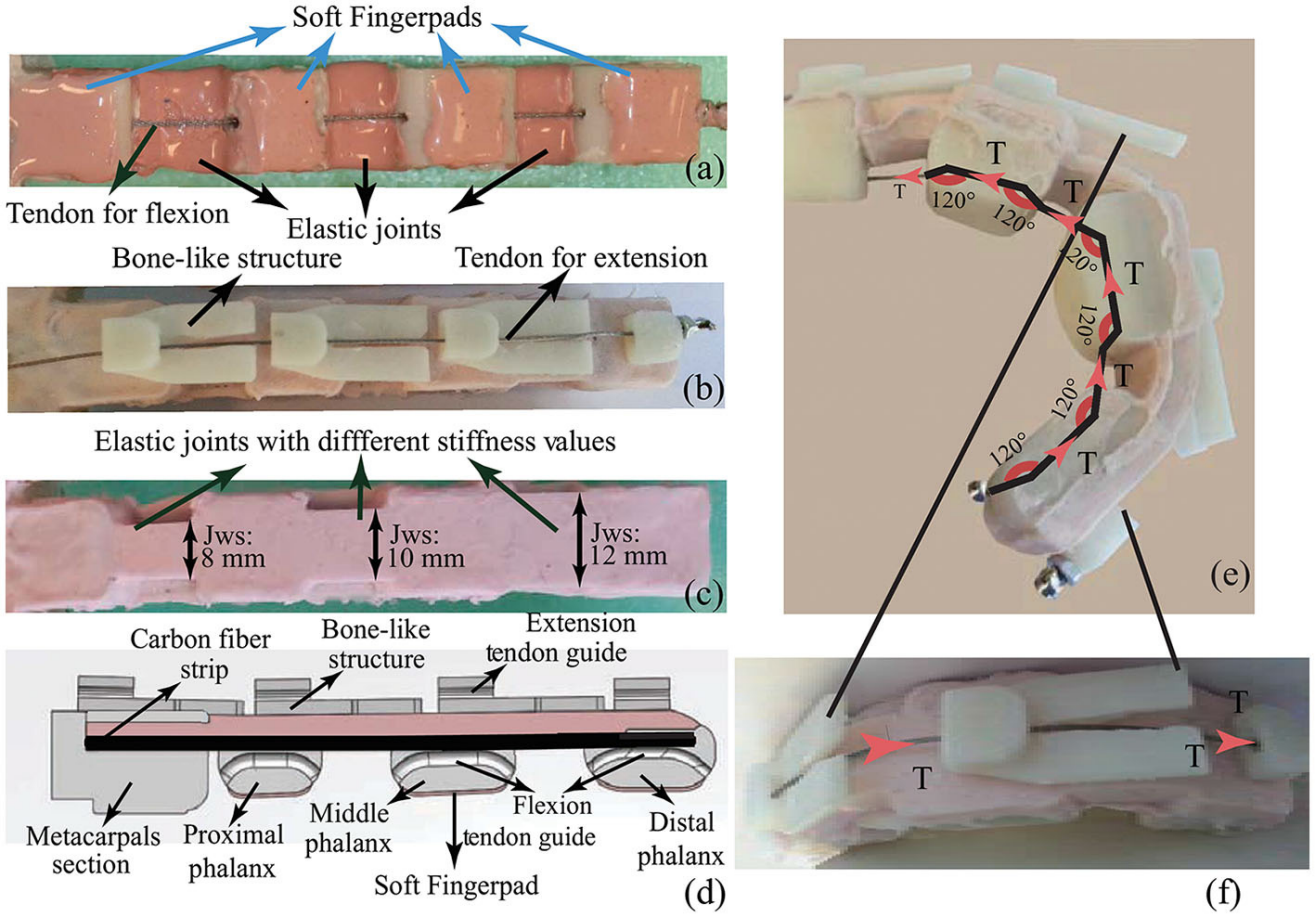
**(a)** Bottom view with soft finger pads. **(b)** Top view with bone-like joint limits. **(c)** Side view with varying joint widths resulting in customized joint stiffness. **(d)** Cross-section of a solid model of the compliant finger with cable routing. **(e)** Flexion tendon routing. **(f)** Extension tendon routing.

In the second step, finger molds are used for the precise arrangement of phalanges, compliant joints, and fingers. Epoxy resin infused carbon fiber sheets are incorporated into the design as in Figure 5b, before injecting silicone rubber in liquid form. This way, even though each joint consists of the same materials, silicone rubber molds with varying widths enable specific stiffness levels to be associated at each joint (refer to Figures 6c,d). In particular, the stiffness is increased from proximal to distal joints. Consequently, the joint flexion initializes at the metacarpophalangeal joint, continues at the proximal interphalangeal joint, and finalizes at the distal interphalangeal joint.

In the third step, highly-adhesive silicone rubber is injected into the mold in its liquid form as depicted in Figure 5c. Before pouring the silicon resin, routing holes at phalanges are strapped in order to prevent polyurethane flow inside tendon routes. In addition, a release agent is used to avoid the bonding of cured polyurethane to mold surfaces and to facilitate the releasing of the part from the crinkled-shaped mold.

As a consequence of injecting silicon rubber into the mold, air bubbles occur inevitably, which may cause inhomogeneous material distribution, adversely affecting the stiffness of each joint; hence, the coordinated motion of the finger. In the fourth step, degassing is implemented with –0.2 to –0.5 bar pressure to remove air bubbles from the silicone material, as presented in Figure 5d. The recommended cure time for the resin is about 12 h at room temperature and the complete molding process takes about 13 h.

In the fifth step, the fingers are removed from the molds and tendons responsible for force transmission are inserted into their routes, as shown in Figure 5e.

In the final step shown in Figure 5f, bone-like structures, as in Figure 6b, are placed on the upper surface of fingers to induce physical joint limits. These structures constrain the finger extension after it reaches its fully extended horizontal position, preventing fingers to bend in the reverse direction. High friction soft finger pads produced using the silicone material are added on the contact surfaces of phalanges to improve slip resistance of the fingers (Cutkosky et al., 1987; Shimoga and Goldenberg, 1992), as in Figure 6a.

##### 2.3.3.3. Cable Routing for the Compliant Fingers

Force transmission of the compliant fingers is achieved through the flexion and the extension tendons. Due to the inherent stiffness of each compliant joint, higher forces are required while closing the fingers. To facilitate easier closing of the fingers, flexion cable channels are implemented with 120° angles, such that larger moment arms are implemented for the flexion, increasing the moments acting on the phalanges. Figure 6d depicts the cross-section of a solid model of a compliant finger, while Figures 6e,f show the flexion and extension tendon routing on a finger prototype.

#### 2.3.4. Implementation of the Transradial Hand Prosthesis

**Figure 7:**
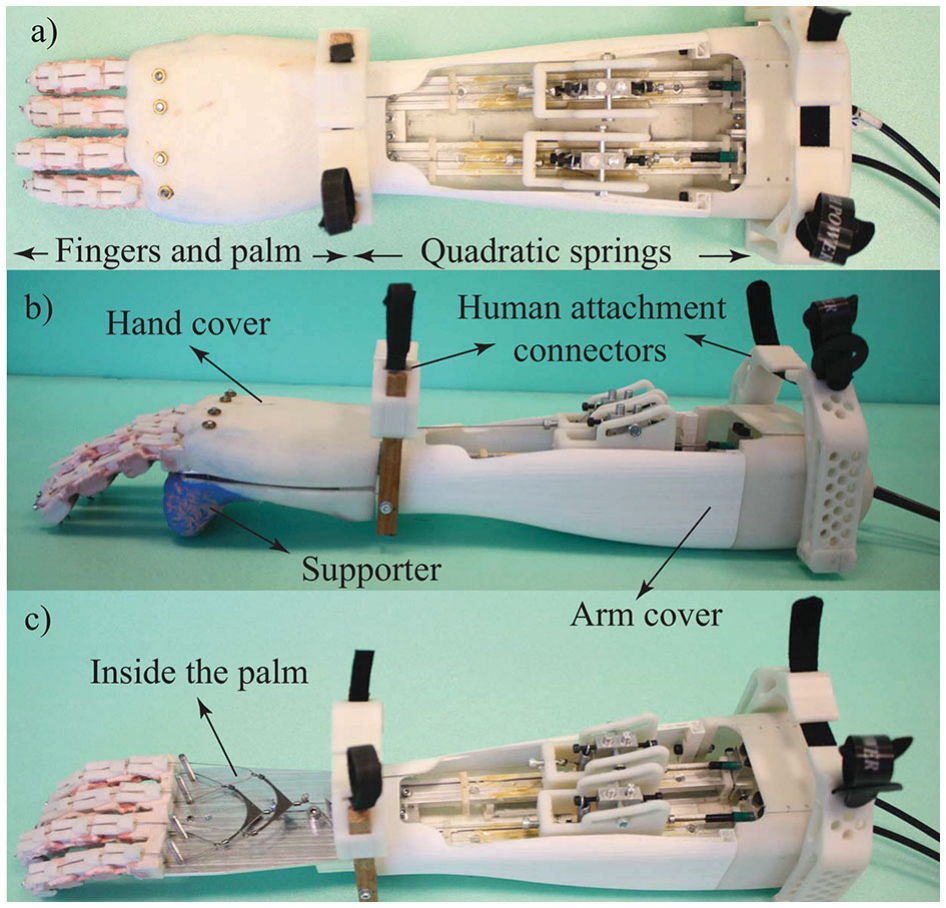
**(a)** Top view, **(b)** side view, and **(c)** interior of the transradial hand prosthesis prototype.

The hand prosthesis consists of three main components: VSA, forearm, and compliant fingers. The expanding contour cams of VSA, the forearm in which the nonlinear springs of VSA are embedded on linear sliders, and device covers are fabricated through additive manufacturing, while the palm is constructed using a laser cut aluminum sheet. Figure 7 presents an assembled prototype.

Since the actuators, motor drivers, and batteries of the Bowden cable driven VSA can be placed remotely anywhere on the body, e.g., can be kept inside a backpack, they are not integrated inside the prosthesis. This decision helps keep the device weight and cost low, as it provides extensive design flexibility while choosing/customizing these components according to the needs of the amputee. Sizing of the actuators should be decided considering the friction induced by Bowden cables. Note that, integration of this component into the forearm is also possible but induces challenging size and weight constraints for these components.

The specifications required from the drive train are characterized as follows. The maximum force required on the drive tendon such that a 1.5 kg object can be lifted at the maximum stiffness level is verified as 160 N. The force required to close the hand at the lowest stiffness level with no object in the hand is characterized as 20 N. The maximum speed required on the drive tendon is characterized as 20 mm/s, such that all fingers reach their joint limits within 2 s when the fingers are free to move.

Geared DC motors that satisfy the maximum force and speed requirements are selected to implement the drive train. With these motors, the minimum and the maximum stiffness of the drive tendon are experimentally characterized as 135 and 545 Nmm/rad, respectively. The overall weight of the device (excluding the motors, drivers, and battery) is 1.1 kg, which is lower than the natural weight of the corresponding part of the human limb (Plagenhoef et al., 1983; de Leva, 1996). Lightweight may cause less fatigue for the amputee. Note that the current research prototype is developed to verify the intended functionality and has not been optimized for size and weight. If necessary, the weight of the prosthesis can easily be adjusted to match the weight of the lost limb. The drive train specifications of the hand prosthesis are presented in Table 1.

**Table 1:** Technical specifications.

Maximum tendon force	160 N
Minimum tendon force	20 N
Maximum tendon speed	20 mm/s
Minimum joint stiffness	135 Nmm/rad
Maximum joint stiffness	545 Nmm/rad
Weight	1.1 kg

To select a properly sized battery pack, the power consumption of the transradial hand prosthesis is experimentally characterized, as it is dependent on the grasp type and the friction losses in the system. Power and pinch grasps are studied as these are most commonly used. Each grasp type is repeatedly executed on a wide variety of objects, when the hand stiffness is set to high and low values, respectively. Furthermore, the average power consumed while modulating the hand stiffness from its lowest level to highest level is also characterized. The mean power consumption for each grasp type and stiffness change are presented in Table 2.

**Table 2:** Power consumption.

	**Energy requirements [mWh]**	
	**Low stiffness**	**High stiffness**
Power grasp	30	81
Pinch grasp	18	75
Stiffness modulation	4.9	

The highest energy consumption takes place during a power grasp when the stiffness is simultaneously modulated from low to high. The results indicate that a 50 g rechargeable LiFePo4 battery pack with 1,500 mAh enables the execution of this grasp for 120 times. Furthermore, these battery modules can be rapidly charged. The number of battery modules integrated into the system can be personalized based on the needs and preferences of the amputee.

## 3. Results

The aim of the experimental evaluation is to reveal the feasibility of the working principle of the proposed hand prosthesis. A set of experiments is conducted in order to validate the independent position and stiffness modulation of the hand prosthesis. The experiment consists of two tasks, where the objective of the first task is to verify stiffness modulation when the position is regulated at a constant value, while the second task aims to verify that the desired position can be changed when the stiffness parameters are kept constant.

### 3.1. Experimental Setup and Procedure

**Figure 8:**
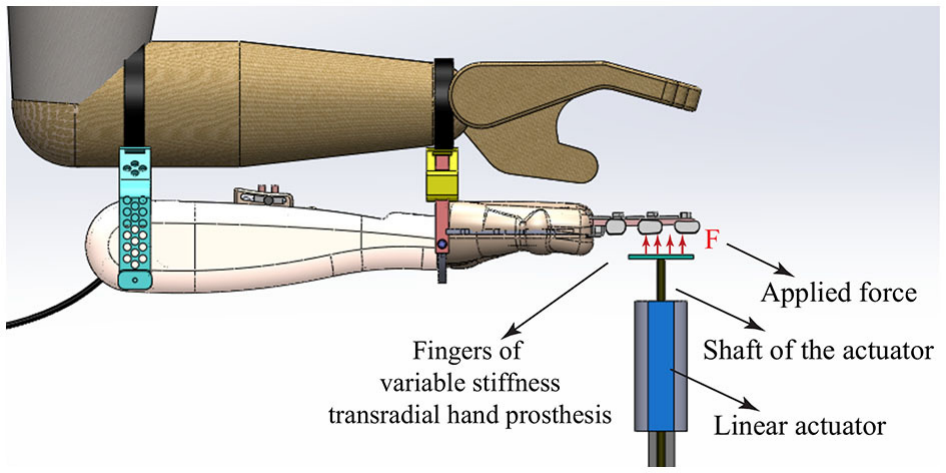
Schematic representation of the experimental setup.

The experimental setup consists of a direct drive linear actuator with a built-in high resolution incremental encoder, placed under the four fingers of the transradial prosthesis, as shown in Figure 8. During the experiments, the gravitational force acting on the actuator is compensated with a counter mass, while the actuator is force controlled. All controllers are implemented in real-time at 500 Hz with a PC workstation equipped with a DAQ card. In these experiments, the position and the stiffness levels are not controlled by the volunteers, as the goal is to perform a verification of the hand prosthesis independent of its user interface. Hence, during the experiments, the reference values for the position and the stiffness are set by the PC workstation.

The experiment is composed of two tasks with 10 repetitions for each condition of each task. During the first task, the position of the VSA is kept constant at 0°, i.e., the angular position of the metacarpophalangeal (MCP) joint is set to 0°, while the stiffness of VSA is adjusted to three distinct stiffness values that correspond to low, intermediate, and high stiffness levels for the fingers. The stiffness of the fingers is experimentally determined by applying a linearly increasing force to flex the fingers and recording their deflection.

During the second task, the stiffness of the VSA is kept constant at its intermediate level, while the position of the VSA is adjusted to three distinct position values that correspond to low, intermediate, and high flexion of the fingers. The position of the fingers is determined by recording the position of the linear actuator under zero force control, while the stiffness of the fingers is determined by applying a constant force to resist flexion of the fingers at the equilibrium position and recording the resulting deflection.

### 3.2. Experimental Results

**Figure 9:**
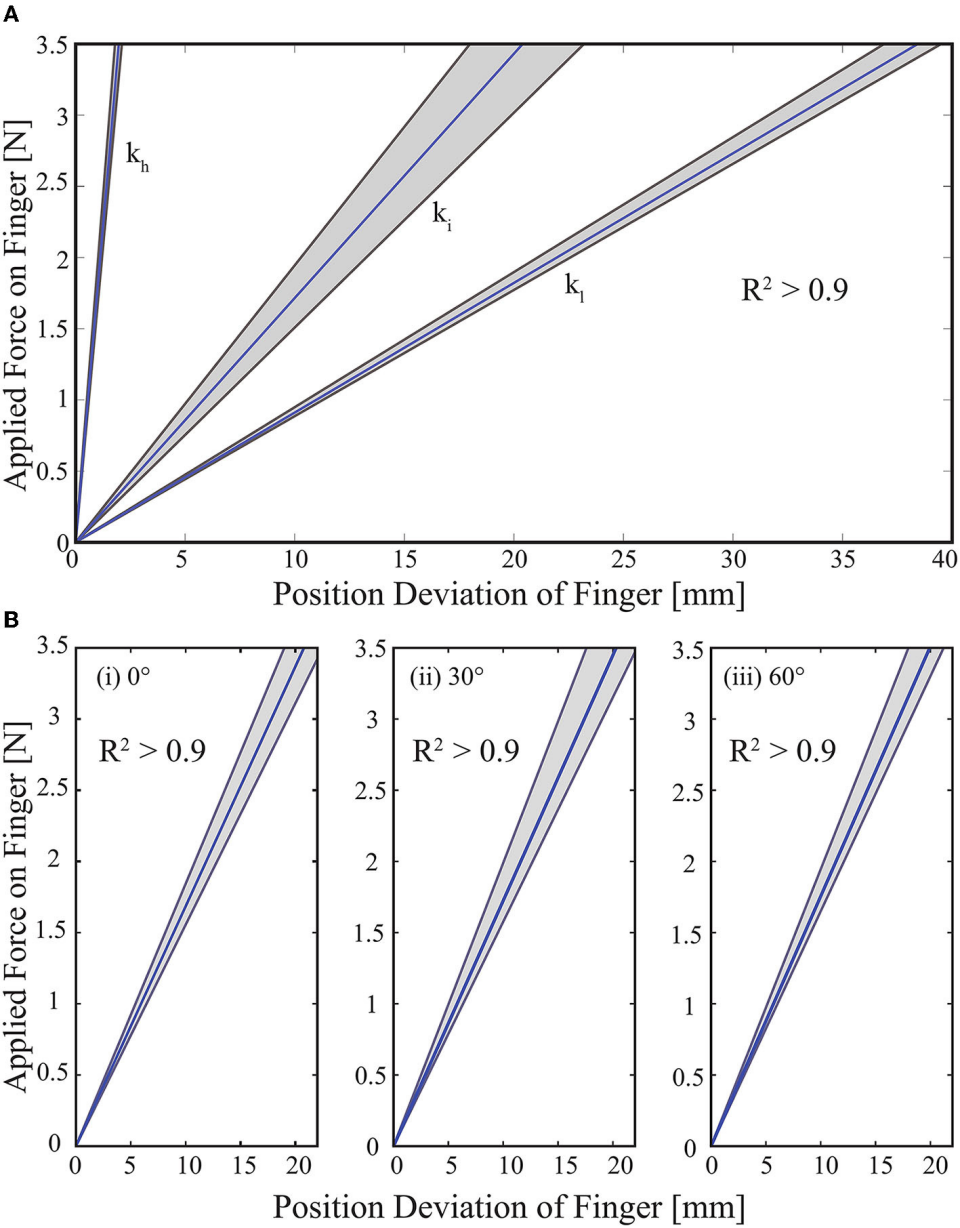
**(A)** Stiffness modulation of hand prosthesis. The Gray zone presents the best linear fit of each trial and the blue line presents the average value of ten trials. **(B)** Position control of hand prosthesis. The Gray zone presents the results of each trial. The blue line presents the average value of ten trials.

Figure 9A presents the experimental results for the case when VSA is adjusted to three distinct stiffness values that correspond to low, intermediate, and high stiffness levels for the fingers, while the finger positions are kept constant. In particular, shaded regions represent all the linear fits recorded for 10 trials, while the dark line represents their mean. The slopes of these lines indicate that low, intermediate, and high stiffness for the fingers are *k*_*l*_=0.091 N/mm, *k*_*i*_=0.17 N/mm, and *k*_*h*_=1.8 N/mm, respectively. The *R* values for these linear fits are evaluated to be higher than 0.98.

Figure 9B presents the experimental results for the case when VSA is adjusted to intermediate stiffness level, while the finger positions, i.e., the angle between the MCP joint of the fingers and the palm surface, are regulated to 0°, 30°, and 60°, respectively. Once again, the shaded regions represent all the linear fits recorded for 10 trials, while the dark line represents their mean. The slopes of these lines indicate that the stiffness level of the fingers are $k_{0^{∙}}$=0.17 N/mm, $k_{30^{∙}}$=0.17 N/mm, and $k_{60^{∙}}$=0.18 N/mm, respectively. The *R* values for these linear fits are evaluated to be higher than 0.98.

Experimental results provide strong evidence that the stiffness and the position of the transradial hand prosthesis can be controlled independently, with high repeatability while executing predefined tasks. The impedance characteristics of the compliant fingers of the VSA prosthesis closely match the characteristics of human fingers as presented in Howe et al. (1985). The characterization results are also compatible with the results presented in Matsuoka and Afshar (2004), as the flexion/extension movements performed by an anatomically human-like robotic index finger necessitate a similar amount of muscle forces.

### 3.3. Illustrative Experiments and Evaluations

Given that only the position and the stiffness of the driven tendon are directly regulated by the VSA, in general, the resulting position and stiffness of the fingers depending on the interaction. To test the usefulness of the variable stiffness transradial hand prosthesis, the device is attached to six volunteers, as shown in Figure 8. All volunteers signed informed consent forms approved by the IRB of Sabanci University. The volunteers were given control of the position and the stiffness of the prosthesis through the sEMG based tele-impedance controller (Hocaoglu and Patoglu, 2012, 2019).

**Figure 10:**
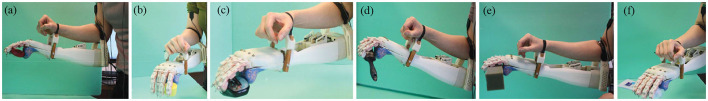
Demonstration of variable stiffness transradial hand prosthesis performing various grasps while interacting with **(a)** a glue box, **(b)** a bottle cap, **(c)** a mouse, **(d)** a brush, **(e)** a sponge, and **(f)** a cream tube.

In particular, sEMG signals measured from the surface of the upper arm, chest, and shoulder were used to automatically adjust the stiffness level of the prosthesis to that of the upper arm, while the position regulation was intentionally controlled by the volunteers by moving their shoulder muscles. With this natural control interface, the volunteers were asked to grasp 16 objects with a wide variety of shapes (e.g., rectangular, elliptic, complex) and compliance levels (e.g., rigid, elastic). Participants were asked to grasp and hold the objects for a while, then release them back onto the surface. In the same manner as in Section 3, the required stiffness level of VSA to safely grasp each object depended on the interaction. The volunteers were successful at modulating their impedance and grasping a wide variety of objects with the sEMG interface, as shown in Figure 10. Videos demonstrating several illustrative grasps are available at https://hmi.sabanciuniv.edu/VSA_hand_prosthesis.mp4.

The current prototype emphasizes simplicity, ease of use, and adaptability; hence, implements a two-degree-of-freedom underactuated power transmission to allow for the position and stiffness change of the prosthetic hand. Successful employment of the prosthesis depends on the amputee making proper decisions on how to interact with the object. Our extensive experiments with volunteers indicate that humans are very skillful at learning how to interact with the environment.

On average, it took 3.2±1.3 min for a volunteer to get used to the device and successfully complete the required manipulation tasks. The time elapsed for grasping and releasing the 15 objects in the video are calculated as 1.22±0.56 s and 0.82±0.48 s, respectively. The time required to make a fist is about 2 s. The grasping performance of the proposed prosthesis prototype is comparable to commercial ones (Touch Bionics Inc., 2022).

The robustness of the hand prosthesis is also tested during the user studies. During the user studies, volunteers repeatedly impacted the fingers to various surfaces. Compliant fingers made of silicon rubber and ABS material were robust to such impacts and did not sustain any damage.

In the current research prototype, passive support is preferred to oppose the fingers, instead of an active thumb. This decision helps keep the system and the controller simple. Our experiences with the volunteers indicate that the passive support is adequate for implementing a wide variety of functional grasps. During post trial interviews, none of our volunteers complained about the functionality of this support.

## 4. Discussion

In this study, the design, implementation, and experimental evaluation of a low-cost, customizable, easy-to-use variable stiffness transradial hand prosthesis have been presented. User studies indicate that the device is dexterous enough to successfully interact with a wide variety of environments.

Please note that the classification of the design objectives, as presented in Section 2.1, is subjective. In general, it is up to the designer to prioritize the users' needs. Based on different prioritization of these objectives, it is possible to come up with different designs, catering to various user types. In our design, the goal has been to achieve a dexterous, anthropomorphic, adaptable, robust, low-cost mechatronic system design. Achieving such a mechatronic system design is only the half of the story toward achieving an ideal prosthesis, where the design of an easy-to-use control interface for the mechatronic system is the other half. We present such a human-machine interface, called *tele-impedance control*, in Hocaoglu and Patoglu (2019).

The goal of our design is to provide a fulfilling functionality to amputees through stiffness modulation while decreasing or even reversing the relatively high abandonment rate for current high-tech prosthetics. While some of the commercial devices provide many extra functions including active thumb and wrist movements, these devices require special training and long rehabilitation periods, which discourage the majority of amputees from continually using such devices. It is possible to add a thumb to the tendon driven power transmission of the proposed prosthesis; however, substantial effort is required for the intricate control of thumb orientation and stiffness, such that it becomes functional and enhances the grasping performance. Alternatively, to achieve an anthropomorphic look with an aesthetically pleasing appearance, a compliant unactuated thumb can be added to the system. Furthermore, prosthesis gloves can be worn over the transradial hand prosthesis to achieve the natural appearance of human skin.

Our ongoing research study includes testing the efficacy of the variable stiffness hand prosthesis and its control interface (Hocaoglu and Patoglu, 2019), and is an extension of Section 3.3. This study has a comprehensive human subject experiment design to compare variable stiffness prostheses with fixed stiffness prosthetic hands. Our preliminary results with healthy volunteers presented in Section 5 of the PhD thesis of the first author (Hocaoglu, 2014), already provide strong evidence that tasks that require stiffness regulation by sEMG signals are performed best with the variable stiffness prosthetic hand in comparison to the performance obtained by a prosthesis whose stiffness level is fixed to a constant value.

Our future study will focus on improving the anthropomorphism and functionality by the inclusion of active thumb, wrist, and auto pre-tension mechanisms to the hand prosthesis, providing a customized sizing and weight distribution of the design, evaluating the grasping performance of the hand prosthesis on the same objects by setting the stiffness of the hand to different levels, conducting comprehensive testing with larger healthy populations and also amputees to provide evidence of the effectiveness of variable stiffness actuation, and refinement of the prosthesis based on the feedback collected from amputees.

## Data Availability Statement

The original contributions presented in the study are included in the article/supplementary material, further inquiries can be directed to the corresponding author.

## Ethics Statement

The studies involving human participants were reviewed and approved by University Research Ethics Council of Sabancı University. The patients/participants provided their written informed consent to participate in this study.

## Author Contributions

EH was responsible for the design, fabrication, and experimental evaluation of the variable stiffness hand prosthesis. VP conceived and supervised the study. Both authors contributed to the editing and scientific presentation of the article.

## Funding

This work has been partially supported by Tubitak Grants 219M586, 111M186, 115M698 and Marie Curie IRG Rehab-DUET.

## Conflict of Interest

The authors declare that the research was conducted in the absence of any commercial or financial relationships that could be construed as a potential conflict of interest.

## Publisher's Note

All claims expressed in this article are solely those of the authors and do not necessarily represent those of their affiliated organizations, or those of the publisher, the editors and the reviewers. Any product that may be evaluated in this article, or claim that may be made by its manufacturer, is not guaranteed or endorsed by the publisher.
